# Analysis of the Effect of Lyophilized Recombinant Human Brain Natriuretic Peptide on Endothelial Function in patients with acute myocardial infarction

**DOI:** 10.12669/pjms.37.1.2706

**Published:** 2021

**Authors:** Bosong Wang, Hong Xu, Chengqin Li, Xiaoding Wang, Weidong Sun, Jinlong Li

**Affiliations:** 1 Bosong Wang, Department of Cardiovascular III, Taian City Central Hospital, Taian, 271000, China; 2 Hong Xu, Department of Cardiovascular III, Taian City Central Hospital, Taian, 271000, China; 3 Chengqin Li, Department of Cardiovascular III, Taian City Central Hospital, Taian, 271000, China; 4 Xiaoding Wang, Department of Cardiovascular III, Taian City Central Hospital, Taian, 271000, China; 5 Weidong Sun, Department of Cardiovascular III, Taian City Central Hospital, Taian, 271000, China; 6 Jinlong Li, Department of Cardiovascular III, Taian City Central Hospital, Taian, 271000, China

**Keywords:** Lyophilized recombinant human brain natriuretic peptide, Acute myocardial infarction, Endothelial function

## Abstract

**Objective:**

This study aims to analyze the effect of lyophilized recombinant human brain natriuretic peptide on the endothelial function of patients with acute myocardial infarction.

**Methods:**

One hundred and thirty-six patients with acute myocardial infarction in our hospital were randomly divided into a control group and an experimental group (68 cases each). The patients in the control group were treated by conventional treatment. The patients in the experimental group were treated with lyophilized recombinant human brain natriuretic peptide besides the conventional treatment. The levels of flow-mediated dilatation (FMD), serum nitric oxide (NO), and endothelin-1 were compared between the two groups before and after treatment.

**Results:**

Before treatment, there was no significant difference between the two groups in the level of FMD (P>0.05); after treatment, the level of FMD in the experimental group was higher than that in the control group, and the difference was statistically significant (P<0.05); before treatment, there was no significant difference between the two groups in the levels of serum NO and endothelin-1 (P>0.05); after treatment, the levels of serum NO and endothelin-1 in the experimental group significantly improved, which were better than those in the control group (P<0.05).

**Conclusion:**

Lyophilized recombinant human brain natriuretic peptide can improve the FMD, increase the content of NO in the blood, and effectively reduce the level of endothelin-1, which is of great significance to improve the endothelial function of patients with acute myocardial infarction and is worth clinical application.

## INTRODUCTION

Acute myocardial infarction is a common clinical disease with a high mortality rate and poor prognosis.^1^,^2^ The pathological mechanism of acute myocardial infarction is that the rupture of atherosclerotic plaque in the coronary artery leads to the formation of thrombus in the coronary artery, which causes acute myocardial ischemia, hypoxia, and necrosis, accompanied by inflammatory cell infiltration.^3^,^4^ At present, antithrombotic drugs and emergency percutaneous coronary intervention (PCI) are the most effective treatments; however, the compression effect of the balloon on blood vessels and local inflammatory reaction during the operation will damage the vascular endothelium in varying degrees, resulting in the impairment of myocardial microvascular function and the formation of micro thrombosis; as a result, there is no myocardial reperfusion after coronary artery recanalization, which is easy to cause myocardial reperfusion injury in patients.^5^,^6^

Vascular endothelium is a relatively active tissue in the human body, which can secrete multiple factors to regulate homeostasis, inflammatory response, and immune response. Natriuretic peptide is a B-type endogenous natriuretic peptide, which is an important hormone secreted by vascular endothelium, and mainly consists of 32 amino acid residues. Natriuretic peptide was first isolated from pig brain by Japanese scholars and widely exists in atrial, ventricular myocytes, and vascular endothelial cells. It has powerful diuretic, natriuretic, and vasodilator functions.^7^,^8^ Natriuretic peptide increases cyclic guanosine monophosphate by binding with its corresponding receptor in the body, thus expanding the arteries and veins of patients, mediating a series of physiological effects, inhibiting the sympathetic nervous system and renin-angiotensin-aldosterone system, effectively reducing myocardial fibrosis, suppressing the expression of tissue factor in vascular endothelial cells, and preventing thrombosis.

Lyophilized recombinant human brain natriuretic peptide has a structure that is close to endogenous natriuretic peptide secreted by the human body, and their physiological effects are similar. A relevant study indicated that lyophilized recombinant human brain natriuretic peptide had a significant effect in improving cardiac function and flow-mediated dilatation (FMD).^9^ Based on the above studies, this study aimed to analyze the influence of lyophilized recombinant human brain natriuretic peptide on the basis of the conventional treatment on the vascular endothelial function of patients with acute myocardial infarction.

## METHODS

One hundred and thirty-six patients with acute myocardial infarction who were admitted to our hospital from June 2017 to December 2018 were selected for this study. The diagnostic criteria of all subjects were in accordance with the diagnosis of acute myocardial infarction formulated by WHO: no remission of chest pain > 30 minutes; myocardial ischemic changes of at least two consecutive leads of electrocardiograph; positive troponin.^9^ There were 76 cases of males and 60 cases of females. They aged 60 to 85 years (average (72.43±9.15) years). As to the distribution of the infarct site, there were 64 cases of anterior wall infarct and 72 cases of inferior wall infarct. Exclusive criteria included having original severe valvular heart disease, connective tissue disease, autoimmune disease, and a history of major surgery or severe trauma within 15 days of treatment, being allergic to related treatment drugs, or having liver and kidney dysfunction and malignant tumors. This study has been approved by the ethical committee of our hospital (dated March 10, 2018), and the included subjects have signed the informed consent.

The selected patients were randomly divided into two groups, 68 cases in each group. The patients in the control group were given conventional treatment. They were given a conventional dose of dual anti-platelet aggregation, heparin or low molecular weight heparin anticoagulation, statins, and nitric acid ester. According to the disease condition, β-receptor blockers, angiotensin-converting enzyme inhibitors, (ACEI) and diuretics were given as well. Patients who had emergency percutaneous coronary intervention indicators were treated with emergency interventional surgery for revascularization. The patients in the lyophilized recombinant human brain natriuretic peptide treatment group were treated with lyophilized recombinant human brain natriuretic peptide besides the conventional treatment. The load was 1.5 μg/kg, and it was intravenously injected at a uniform rate for 90 s, followed by intravenous injection at a rate of 0.0075-0.0150 μg/(kg·min) for 72 h. The heart rate and blood pressure of both groups were monitored closely in the course of medication.

The vascular endothelial function of the two groups before and two weeks after treatment was detected and compared, mainly through the high-resolution color Doppler blood flow diagnosis system and high-frequency ultrasound probe. The brachial artery at about 2-5 cm above the right elbow of the patient was selected for detection. The end-diastolic diameter of the patient was measured, and the average value of three cardiac cycles was calculated. The basic value (D0) was measured in the supine position after resting for about 10 minutes. In the reactive hyperemia test, the cuff of the sphygmomanometer was inflated and pressurized on the forearm of the patient to 300 mm Hg (1 mm Hg=0.133 kPa) and deflated for 5 min. The brachial artery diameter was measured within 60-90 s after deflation (D1), FMD = [(D1-D0)/D0] × 100%.^10^

The levels of serum NO and endothelin-1 were tested before and two weeks after treatment. The fasting venous blood of the two groups was extracted before and 2 weeks after treatment, and 4 mL of the fasting venous blood was centrifuged at 3500 rpm. After 10 minutes, the serum was separated and stored in a refrigerator at -70^o^C. The levels of serum NO and endothelin-one of the two groups were measured using enzyme-linked immunosorbent assay (ELISA) before and after treatment and compared.

### Statistical Analysis

SPSS23.0 software was used for statistical analysis of data in this study. The measurement data were expressed by mean ± standard deviation; the comparison between the two groups was performed using the t-test. The enumeration data were expressed by %; the comparison between the two groups was performed using the Chi-square test. P<0.05 indicated that the difference was statistically significant.

## RESULTS

There was no statistical difference in the baseline data between the two groups (P>0.05); hence the results were comparable, as shown in Table-I.Before treatment, there was no significant difference in the FMD level between the two groups (P>0.05); after treatment, the FMD level of the experimental group was significantly higher than that of the control group, and the difference was statistically significant (P<0.05) (Table-II). There was no significant difference between the two groups before treatment (P>0.05). After treatment, the level of serum NO in the experimental group was significantly higher than that in the control group, and the level of endothelin-1 was significantly lower than that in the control group (P<0.05) (Table-III).

**Table-I:** Baseline data between the two groups.

Group		Experimental group	Control group	t/X^2^	P

Gender	Male	36	40	0.038	>0.05
	Female	32	28	
Age (years)		73.21±8.40	71.80±9.21	0.768	>0.05
Body mass index (kg/m^2^)		23.87±1.52	24.12±1.80	0.571	>0.05
Location of myocardial infarction	Inframyocardial infarction	38	34	0.796	>0.05
	Anterior myocardial infarction	28	32	
History of disease	Diabetes	18	17	0.251	>0.05
	Hypertension	12	13	0.352	>0.05
	Smoking	19	21	0.054	>0.05
	Drinking	18	20	0.183	>0.05

**Table-II:** FMD between the two groups before and after treatment (%).

Group		Experimental group	Control group	t	P
FMD	Before treatment	7.24±1.51	7.35±1.42	0.486	>0.05
	After treatment	9.15±2.85	15.13±1.17	14.947	<0.05

**Table-III:** Serum NO and endothelin-1 levels between the two groups before and after treatment.

Group		Experimental group	Control group	t	P
Serum NO (μmol/L)	Before treatment	35.86±5.13	36.37±5.52	0.536	>0.05
	After treatment	45.09±5.67	37.53±5.41	7.459	<0.05
Endothelin 1 (ng/L)	Before treatment	83.25±24.15	84.52±23.06	0.295	>0.05
	After treatment	72.15±21.42	82.04±23.05	2.435	<0.05

## DISCUSSION

From the perspective of the pathogenesis of acute myocardial infarction, vascular endothelial dysfunction is an important factor that can not be ignored. Endothelial dysfunction is likely to cause damages to the regulation function of coronary artery tension, resulting in vascular wall remodeling and the activation of platelets and monocytes. Vascular endothelial dysfunction can not only trigger early atherosclerosis but also plays an important role in the process of rupture of plaque.^10^,^11^ As a very active metabolism tissue, vascular endothelium can secrete a variety of factors and participate in body activities, such as body balance regulation, immune response, and inflammatory response.^12^,^13^ Natriuretic peptide, a major hormone secreted by vascular endothelium, is a B-type natriuretic peptide. In the process of natriuretic peptide binding with related receptors, cyclic guanosine monophosphate significantly increases, making smooth muscle cells relax. Cyclic guanosine monophosphate can dilate arteries and veins and mediate a series of physiological effects, such as natriuretic, diuretic, and vasodilator. It can inhibit the activities of the human sympathetic nervous system and renin-angiotensin-aldosterone system and significantly reduce myocardial fibrosis and the expression of tissue factors in vascular endothelial cells, avoiding thrombosis. At present, lyophilized recombinant human brain natriuretic peptide has similar biological effects with endogenous natriuretic peptide and has been widely used in the clinical treatment of diseases, such as heart failure, acute coronary syndrome, and essential hypertension. The unique mechanism of action of lyophilized recombinant human brain natriuretic peptide may provide greater benefits.

It has been reported that the impairment of coronary artery endothelial function is the initial inducement of acute myocardial infarction.^14^ Vascular endothelial function can maintain the stability of the vascular environment and reduce platelet aggregation and thrombosis rate. When heart failure occurs in patients with acute myocardial infarction, the body’s oxidative stress response is enhanced. NO is a free radical, which is one of the participants of redox. It can enhance the activity of the plasminogen activator and expand blood vessels;^15^ endothelin-1 is a vasoconstrictor, which can reflect the damage degree of vascular endothelium.^16^

This study treated patients in the experimental group with lyophilized recombinant human brain natriuretic peptide and obtained significant efficacy. The results showed that there was no significant difference in serum NO and ET-1 levels between the two groups before treatment (P>0.05); after treatment, the level of serum NO in the experimental group was significantly higher than those in the control group, and the level of endothelin-1 was significantly lower than that in the control group (P<0.05), suggesting that lyophilized recombinant human brain natriuretic peptide could promote the synthesis of NO, inhibit the production of endothelin-1, and effectively improve the vascular endothelial function of patients, and the findings were similar to previous research results.^17^,^18^

Another main manifestation of vascular endothelial function is FMD. The detection of FMD in the brachial artery can indirectly reflect the endothelial function of the coronary artery. The technology of high-resolution ultrasound measurement of brachial artery FMD is a non-invasive, convenient, and repeatable quantitative detection technology developed in recent years.^19^ Atherosclerosis is a diffuse disease. Measuring FMD can be used as a “window” to reflect the endothelial function damage of the diseased vessels. The research results of Quan showed that the FMD level of patients who were treated by lyophilized recombinant human brain natriuretic peptide increased significantly.^20^ The results of this study also showed that FMD in the experimental group was significantly higher than that in the control group, indicating that there was vascular endothelial dysfunction in patients with acute myocardial infarction. Vascular endothelial dysfunction is a systemic process. Reflecting vascular function changes by the detection of FMD can help understand the situation of atherosclerosis more comprehensively.

### Limitations of the study

In this study, as the long-term follow-up was not carried out on the patients, the follow-up incidence of cardiac insufficiency and mortality were not statistically recorded; therefore, it is necessary to expand the sample for long-term follow-up study.

## CONCLUSION

To sum up, lyophilized recombinant human brain natriuretic peptide can increase the NO content in the blood of patients with acute myocardial infarction to a certain extent and effectively reduce the level of endothelin-1, which is of great significance to improve the endothelial function of patients with acute myocardial infarction. However, the specific mechanism of action is still unclear, which needs more research and analysis.

### Author’s Contribution:

**BSW & HX:** Study design, data collection and analysis.

**CQL, XDW & WDS:** Manuscript preparation, drafting and revising.

**BSW & JLL:** Review and final approval of manuscript.

**BSW:** Responsible and accountable for the study.
